# Clay nanosheet-mediated delivery of recombinant plasmids expressing artificial miRNAs via leaf spray to prevent infection by plant DNA viruses

**DOI:** 10.1038/s41438-020-00400-2

**Published:** 2020-11-01

**Authors:** Qili Liu, Yanpeng Li, Kedong Xu, Dongxiao Li, Haiyan Hu, Feng Zhou, Puwen Song, Yongang Yu, Qichao Wei, Qian Liu, Weipeng Wang, Ruifang Bu, Haili Sun, Xiaohui Wang, Jianjun Hao, Honglian Li, Chengwei Li

**Affiliations:** 1 Postdoctoral Research Base, Henan Institute of Science and Technology, Xinxiang, China; 2 College of Plant Protection, Henan Agricultural University, Zhengzhou, Henan China; 3 Henan Engineering Research Center of Crop Genome Editing, Henan Institute of Science and Technology, Xinxiang, China; 4 State Key Laboratory of Pulp & Paper Engineering, South China University of Technology, Guangzhou, China; 5 Key Laboratory of Plant Genetics and Molecular Breeding, Zhoukou Normal University, Zhoukou, China; 6 School of Food and Agriculture, The University of Maine, Orono, ME 04469 USA

**Keywords:** Genetic transduction, RNAi

## Abstract

Whitefly-transmitted begomoviruses are economically important plant pathogens that cause severe problems in many crop plants, such as tomato, papaya, cotton, and tobacco. *Tomato yellow leaf curl virus* (TYLCV) is a typical monopartite begomovirus that has been extensively studied, but methods that can efficiently control begomoviruses are still scarce. In this study, we combined artificial microRNA (amiRNA)-mediated silencing technology and clay nanosheet-mediated delivery by spraying and developed a method for efficiently preventing TYLCV infection in tomato plants. We designed three amiRNAs that target different regions of TYLCV to silence virus-produced transcripts. Three plant expression vectors expressing pre-amiRNAs were constructed, and recombinant plasmid DNAs (pDNAs) were loaded onto nontoxic and degradable layered double hydroxide (LDH) clay nanosheets. LDH nanosheets containing multiple pDNAs were sprayed onto plant leaves. We found that the designed amiRNAs were significantly accumulated in leaves 7 days after spraying, while the pDNAs were sustainably detected for 35 days after the spray, suggesting that the LDH nanosheets released pDNAs in a sustained manner, protected pDNAs from degradation and efficiently delivered pDNAs into plant cells. Importantly, when the LDH nanosheets coated with pDNAs were sprayed onto plants infected by TYLCV, both the disease severity and TYLCV viral concentration in sprayed plants were significantly decreased during the 35 days, while the levels of H_2_O_2_ were significantly increased in those plants. Taken together, these results indicate that LDH nanosheets loaded with pDNAs expressing amiRNAs can be a sustainable and promising tool for begomovirus control.

## Introduction

Whitefly-transmitted begomoviruses are economically important plant pathogens that cause severe problems in many crop plants, such as tomato, papaya, cotton, and tobacco^1^. *Tomato yellow leaf curl virus* (TYLCV) is one of the most important viral pathogens of tomatoes. It belongs to the genus *Begomovirus* and is distributed worldwide, being the 3rd most important plant virus^1,2^. Begomoviruses have circular and single-stranded DNA molecules encapsulated in a twinned (quasi)-icosahedral virion^2^. They are classified into two groups based on their genome organization: monopartite, having a single genome component, and bipartite, having two genome components, DNA-A and DNA-B^3^. DNA-A and DNA-B are 2.7–2.8 kb in size, and each component has its own open reading frames (ORFs) oriented in a bidirectional fashion. Monopartite begomoviruses (or DNA-A of bipartite begomoviruses) have six ORFs, with two in the virion sense orientation (AV1/V1 and AV2/V2, encoding the coat protein (CP) and the V2/AV2 protein, respectively) and four in the complementary orientation (AC1/C1–AC4/C4, encoding the replication associated protein (Rep), or C1/AC1 protein; the transcriptional activator protein (TrAP), or C2/AC2 protein; the replication enhancer protein (REn), or C3/AC3 protein; and the C4/AC4 protein)^3^. TYLCV is a monopartite begomovirus.

Managing plant viruses is difficult because no chemicals are highly effective in suppressing viral infection in plants. In recent years, interest in gene silencing mediated by artificial microRNAs (amiRNAs) has increased^4–7^. In this technology, an amiRNA is designed to target genes responsible for viral replication, transmission, or symptom development and interferes with the multiplication and spread of viruses in host plants^4^. amiRNAs are single-stranded (~21 nt long) and are designed by replacing a mature microRNA (miRNA) sequence with a duplex structure within pre-miRNAs^4^. Owing to certain attributes, such as their uniqueness, effectiveness and precision, gene silencing through amiRNAs is considered a second-generation technology of RNA interference (RNAi)^5^. It is easy to optimize single amiRNA sequences to target one or several mRNAs without disturbing the expression of other genes^6,7^.

Delivery of genetic materials and regeneration of transformed plants are very important for genetic transformation of plants, the latter being highly impacted by the liability and stability of the delivery method^8,9^. Methods such as electroporation, biolistics, agrobacterium-mediated delivery, and cationic delivery typically require the regeneration of genetically modified progeny plants. Furthermore, agrobacterium-mediated delivery introduces another pathogen into plants. These methods are not perfect and need improvement in applications^10–13^. Therefore, there is a need to develop more effective, simple, and economical methods.

To enhance the stability, translation efficiency, and cell targeting of a naked mRNA, nanomaterials have been used as effective transportation tools in the genetic engineering of plants^14–16^. Particularly, they have been applied for delivering amiRNAs into plants^17–19^. In this way, dsRNAs can be transferred into plant leaves^20^, roots^21,22^ and flower buds^23^. Mitter et al. reported that a single spray of dsRNA loaded on a designed, nontoxic, and degradable layered double hydroxide (LDH) (BioClay) can protect plants against *pepper mild mottle virus* (PMMoV) and *cucumber mosaic virus* (CMV), which greatly prolongs the protection of plant leaves from 7 to 20 days^20^. dsRNA can enter plants and has a slow release of the RNAs carried with LDH delivery^20^. Recombinant DNA plasmids (pDNAs) are often used in plant genetic engineering, but the delivery of pDNA is an important element affecting the efficiency of plant transformation. The larger and sterically hindered pDNA brings new challenges for the loading and delivery of biomolecules with LDH. The aims of this study were to, for the first time, use LDH nanosheets as a delivery system for transporting recombinant pDNAs into plants and examine the effect of TYLCV suppression in plants.

## Results

### amiRNA design and preparation of pDNA–LDH nanosheets

To improve amiRNA-mediated resistance in plants against TYLCV, three TYLCV-specific amiRNAs were designed based on the conserved region of the AV1 gene and a partial region of the AV2 gene. The designed amiRNAs were amiR-AV1/AV2-1, amiR-AV1-2, and amiR-AV1-3, which targeted three different regions of the AV1 gene (Table S1). AmiR-AV1/AV2-1 targeted the 5′ end of the AV1 transcript and the middle region of the AV2 transcript, as these two genes overlap each other with two different reading frames. AmiR-AV1-2 and amiR-AV1-3 targeted the middle and downstream regions of the AV1 transcript, respectively. No potential off-target effects of these amiRNAs were observed within the available tomato genome database. All three pre-amiRNAs (Table S3) were detected in the cloned plasmid pBI121 using PCR and sequencing (Fig. S1). The structures of the pre-miR159a backbone were predicted, including those for pre-amiRNA-1, pre-amiRNA-2, and pre-amiRNA-3 (Fig. S2).

To understand how LDH supported pDNA on a pDNA–LDH nanosheet, both LDH and pDNA–LDH were analyzed. LDH nanoparticles, which are made up of positively charged brucite-like layers, were synthesized by a microwave-assisted aqueous coprecipitation method. Energy-dispersive X-ray spectrometry (EDS) analysis gave an approximate chemical formula of Mg16.01Al6.44(OH)12.78 (NO_3_)2.07(CO_3_)11.69•9.73H_2_O (Fig. S3). As measured by dynamic light scattering, the LDH nanoparticles possessed a positive zeta potential of +44.9 mV. The LDH nanoparticles exhibited a narrow size distribution with an average hydrodynamic diameter of 35.43 ± 0.07 nm (Z-average particle size) and polydispersity index (PDI) of 0.24, which were much smaller than those of LDHs prepared by hydrothermal treatment (102.56 ± 1.01 nm, with a PDI of 0.16), suggesting that microwave-assisted treatment is a promising way to reduce the preparation time and maintain the necessary morphology.

X-ray diffraction and Fourier transform infrared (FTIR) spectrum analyses of the collected nanomaterials showed typical features of MgAl–LDH-type materials. The X-ray diffraction patterns of LDH samples (Fig. S4a) displayed a series of sharp peaks from basal diffractions at 10.7°, 22°, and 34° corresponding to planes (003), (006), and (009), indicating good crystallinity in the typical layered structure. The basal spacing (0.82 nm) was slightly larger than that reported in the literature, which indicated a greater chance of interlayer ion exchange. Based upon the full width at half maximum at the (003) plane and the Scherrer equation, the thickness of the pristine LDH nanoparticles was calculated to be 6.08 nm. FTIR spectra of LDH samples displayed characteristic absorption bands at 3486 and 1646 cm^−1^, which were attributed to stretching vibrations of O–H and bending vibrations of hydroxyl groups of the interlayered water molecules (Fig. S4b). The peak at 673 cm^−1^ was assigned to metal–oxygen–metal and metal–O–H stretching. Similar to previous observations, the absorption band at 1376 cm^−1^ was the asymmetric stretching vibration mode of C–O in carbonate, which overlapped the signal of the stretching vibration of NO_3_^−^. The morphological features of LDH samples were characterized by scanning electron microscopy (SEM) and transmission electron microscopy (TEM). SEM images showed that the LDH nanoparticles had lateral dimensions varying from 30 to 60 nm while maintaining a narrowly distinguished polygonal platelet-like nanosheet morphology (Fig. S4c). Most sheets were well formed in a hexagonal shape (Fig. S4d, e).

**Fig. 1:**
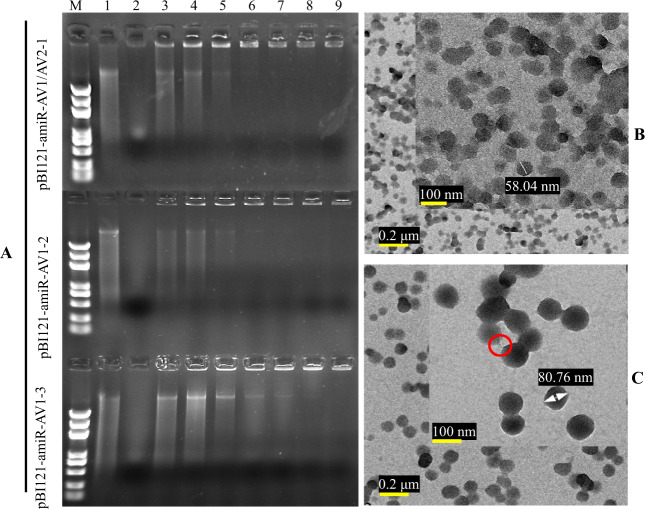
Characterization of layered double hydroxide (LDH) nanosheets and loaded pDNA. **a** pDNA at pDNA:LDH mass ratios of 1:1, 1:2, 1:4, 1:8, 1:10, 1:12, and 1:16, corresponding to lanes 3, 4, 5, 6, 7, 8, and 9, respectively. pDNA only (lane 1) and LDH only (lane 2). *M* = 5 kb DNA ladder, LDH-bound pDNA was detected by fluorescence in wells. Complete loading was achieved at pDNA–LDH mass ratios of 1:10 (lane 7) and 1:12 (lane 8). **b** Transmission electron microscopy (TEM) images of the LDH structure. **c** TEM images of the pDNA–LDH nanocomposite. The surface of the pDNA–LDH composites was smooth, and the nanoparticles tended to have a round shape. The red circle shows a part of the structure of pDNA between LDH nanosheets. Dose ratio = 1:1, *T* = 298 K

Successful formation of a pDNA–LDH complex was confirmed by negligible migration from wells during electrophoresis (Fig. 1a). The constructed pDNA pBI121-amiR showed complete loading at pDNA:LDH mass ratios of 1:10 (pBI121-amiR-AV1/AV2-1 and pBI121-amiR-AV1-2, Fig. 1a lane 7) and 1:12 (pBI121-amiR-AV1-3, Fig. 1a lane 8). This indicated that LDH nanosheets bound purified pDNA molecules ∼13 kb in length. Compared to pristine LDH nanomaterials (Fig. 1b), pDNA–LDH composites were larger (average ~80 nm), while the surface of pDNA–LDH composites were smoother, and the nanoparticles mostly showed a round shape (Fig. 1c). A part of the structure of pDNA was also observed between LDH nanoparticles (Fig. 1c).

### Stability of pDNAs on LDH nanosheets

**Fig. 2:**
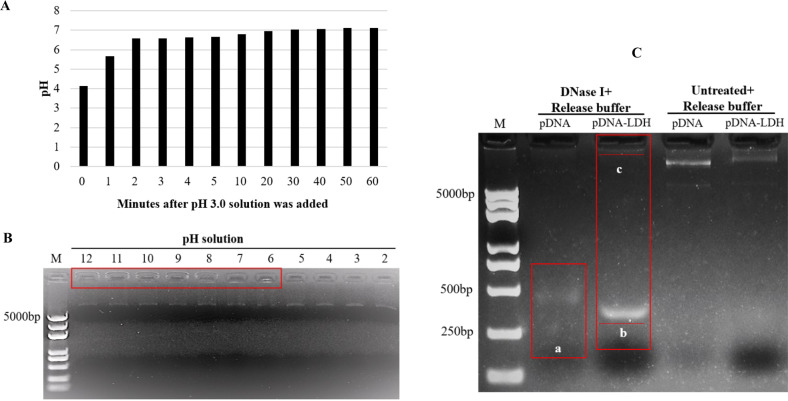
Stability of pDNA on layered double hydroxide (LDH) nanosheets examined by acidic and enzymatic treatments. **a** pH value dynamics over 60 min from the introduction of diluted nitric acid (pH 3.0) to LDH solution. The pH increased from acidic to basic values within 60 min from the introduction of diluted nitric acid (pH 3.0) to LDH solution. The initial pH of 3.0 quickly changed in the first several minutes and stabilized at pH 7.12 after 1 h, indicating dissolution of LDH under acidic conditions. **b** pDNA–LDH was suspended in solutions at a range of initial pH values (2.0–12.0) and incubated for 24 h. pDNA release was complete in pH ≤ 5.0 solutions. At pH ≥ 6.0, most of the pDNA loaded on LDH nanosheets did not migrate on the gel during electrophoresis, as indicated by the florescence being retained in the well. *M* = 5 kb DNA ladder. **c** Treatment of pDNA and pDNA–LDH with DNase I. Gel electrophoresis showed that the treated pDNA was released from the LDH nanosheets and almost completely degraded (a). The treated pDNA–LDH showed that a part of the DNA was released from the LDH nanosheets and incompletely degraded (b). The other pDNA was released from the LDH nanosheets without degradation, which was indicated by the florescence being retained near the well (c). *M* = 5 kb DNA ladder. pDNA–LDH: pDNA loaded on LDH nanosheets, pDNA: plasmid DNA

For pDNA–LDH to provide prolonged pDNA-mediated protection against viruses, pDNA must not only remain on the leaf surface for a longer period than naked pDNA but also withstand field conditions, such as rain, sunlight, and nucleases. Atmospheric CO_2_ and moisture can slowly breakdown LDH into biocompatible residue (Mg^2+^, Al (OH)_3_, and NO^3−^, and so on), releasing the loaded biomolecules^24^. To evaluate the delivery and release of pDNA bonded on LDH nanosheets, the stability of pDNA–LDH was examined for delivering and releasing pDNA in acidic conditions. The pH increased from acidic to basic values within 60 min from the introduction of diluted nitric acid (pH 3.0) to LDH solution. The initial pH of 3.0 quickly changed in the first several minutes and stabilized at pH 7.12 after 1 h, indicating dissolution of LDH under acidic conditions (Fig. 2a). pDNA–LDH was resuspended in solutions at a range of initial pH values (2.0–12.0) and incubated for 24 h. Complete release of pDNA was observed at pH 2.0, 3.0, 4.0, and 5.0, which was reflected by no florescence being retained in the well (Fig. 2b). At pH ≥ 6.0, most of the pDNA loaded on LDH nanosheets did not migrate on the gel during electrophoresis, as indicated by the florescence being retained in the well (Fig. 2b).

The ability of the LDH nanosheets to protect loaded pDNA from potential environmental breakdown, such as enzymatic conditions, was investigated by exposure of pDNA and pDNA–LDH to DNase I. Gel electrophoresis showed that the treated pDNA was released from the LDH nanosheets and almost completely degraded (Fig. 2c–a). The treated pDNA–LDH showed that some of the DNA was released from the LDH nanosheets and incompletely degraded (Fig. 2c–b), while the other pDNA was released from the LDH nanosheets without degradation, which was indicated by the florescence being retained near the well (Fig. 2c–c). Accordingly, DNase I treatment degraded the naked pDNA to a greater extent than the pDNA bonded to LDH nanosheets, suggesting that the LDH nanosheets protected the pDNAs from DNase I degradation.

### Delivery of pDNAs into plant cells and amiRNA expression

**Fig. 3:**
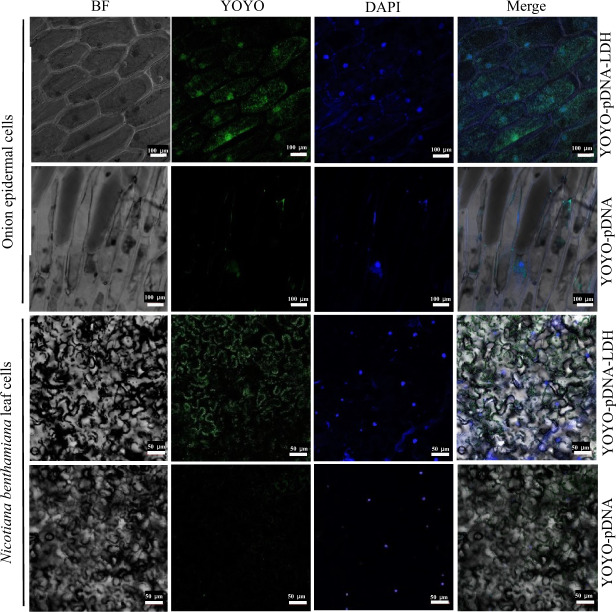
The delivery efficiency of foliar application of pDNA-LDH into plant cells. Cells of onion epidermis and *Nicotiana benthamiana* leaves were observed under a confocal microscope, with bright field (BF), YOYO florescence, DAPI stain and merged images shown for samples at 72 h after treatment with YOYO–pDNA–LDH and YOYO–pDNA. YOYO–pDNA–LDH: pDNA loaded on LDH nanosheets and labeled with YOYO, YOYO–pDNA: pDNA labeled with YOYO

To examine the efficiency of foliar application of pDNA–LDH, YOYO-1 was used to label the nanosheets for visualization in plant cells. Once the pDNA/YOYO-1 complexes were formed, they were stable and exhibited a very high affinity between dye and the pDNA. The results indicated that cells of onion epidermis and *Nicotiana benthamiana* leaves treated with YOYO–pDNA–LDH showed an abundance of YOYO in the nucleus or membranes after 72 h post treatment (Fig. 3).

Systemic movement of YOYO florescence occurred from the outside to the inside of the plant cells in the YOYO–pDNA–LDH-treated plants. These observations suggested that pDNA can be taken up by plant cells with the help of LDH nanosheet transport and release. Moreover, at 35 days after spraying, the pBI121 vector was detected from nearby unsprayed new leaves, which indicated that the pDNA–LDH was delivered systemically from sprayed leaves to unsprayed new leaves in the treated *N. benthamiana* and *Solanum lycopersicum* plants (Fig. S5).

**Fig. 4:**
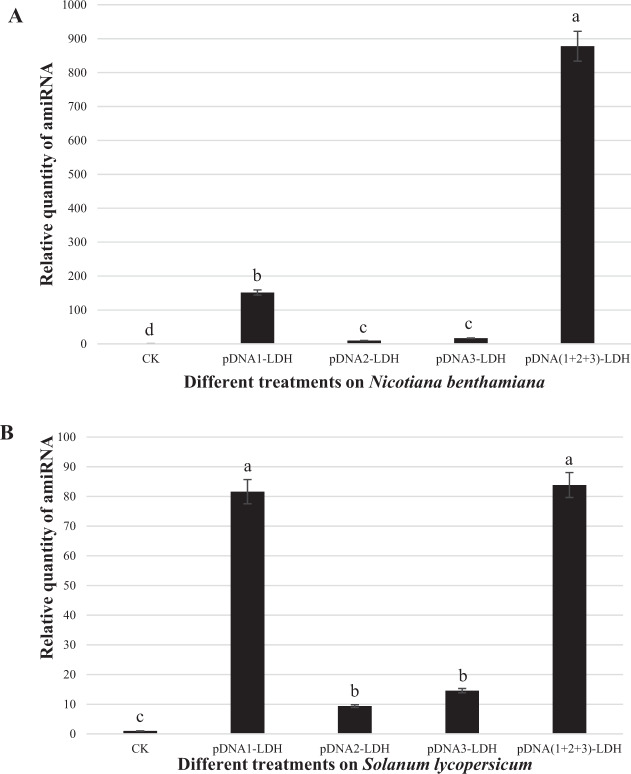
Expression of mixed mature amiRNAs for diverse treatments. Expression levels of mixed mature miRNAs in *Nicotiana benthamiana* (**a**) and *Solanum lycopersicum* (**b**) after they were sprayed with pBI121–LDH (CK), pDNA1–LDH (amiRNA-AV1/AV2-1), pDNA2–LDH (amiRNA-AV1-2), pDNA3–LDH (amiRNA-AV1-3), and pDNA(1 + 2 + 3)-LDH (mixed with three pDNA–LDH), as determined by qRT-PCR assays. The bars for each column represent standard deviations. Values (means ± SEs) are the averages of three independent experiments, and significant differences (*P* ≤ 0.05) among treatments in each group are indicated by different letters

Expression of mixed mature amiRNAs was characterized by qRT-PCR using a Mir-X™ miRNA First-Strand Synthesis kit (TaKaRa Bio, USA) for diverse treatments of *N. benthamiana* and *S. lycopersicum* to investigate their role in suppressing TYLCV. In *N. benthamiana* treated with multiple pDNA-LDHs, amiRNA expression was upregulated to levels approximately seventy times that of the control group and greater than those of the other single pDNA–LDH treatments (Fig. 4a). In *S. lycopersicum* treated with multiple pDNA-LDHs, amiRNA expression was approximately one hundred and ninety times that of the control group (Fig. 4b).

### Preventing TYLCV by foliar spray of pDNA–LDH

**Fig. 5:**
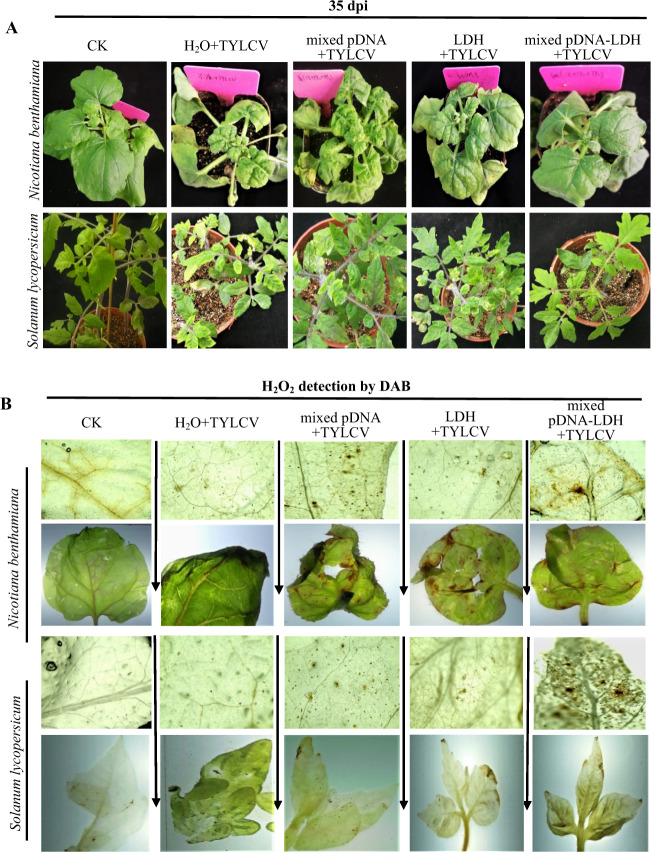
Effect of foliar treatments on the *tomato yellow leaf curl virus* (TYLCV) control evaluated at 35 days post-TYLCV inoculation. **a** Lesions on *Nicotiana benthamiana* (upper panel) and *Solanum lycopersicum* (lower panel) leaves. **b** Plant leaves stained with 3,3-diaminobenzidine (DAB). Treatments included H_2_O plants were sprayed with distillation–distillation water, pDNA plants were sprayed with mixed pDNA, LDH plants were sprayed with LDH, and pDNA–LDH plants were sprayed with mixed pDNA–LDH

Thirty minutes after foliar spraying, leaf cells and stomata of *N. benthamiana* and *S. lycopersicum* plants were not significantly different between pDNA–LDH, pDNA, and control treatments (Fig. S6). At 35 days post-TYLCV inoculation, the infectivity of TYLCV in *N. benthamiana* and *S. lycopersicum* plants harboring amiRNA was significantly suppressed compared to those of plants receiving the other treatments (Table S2). Disease incidence and severity were both lower in mixed pDNA–LDH-treated plants than in nontreated plants (Fig. 5a). All plants inoculated with TYLCV showed viral symptoms that spread from the top of the young leaves but the appearance times of symptoms in mixed pDNA–LDH-treated plants were significantly delayed (Fig. 5a). The symptom expression rate of mixed pDNA–LDH-treated *S. lycopersicum* plants was 41.7% (Table S2) over up to 35 days after inoculation, which was significantly lower than that for other treatments and closely related to the high expression of mixed mature amiRNAs (Fig. 4).

**Fig. 6:**
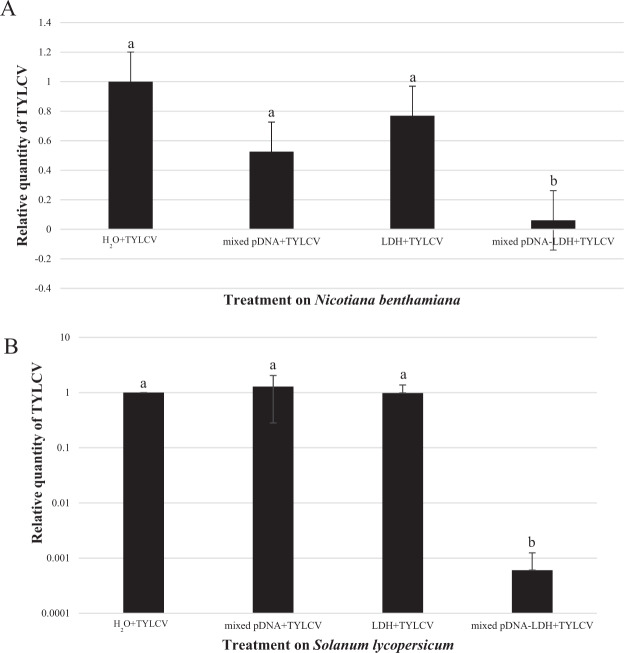
Quantities of tomato yellow leaf curl virus (TYLCV) for diverse treatments. Quantities of *tomato yellow leaf curl virus* (TYLCV) in leaves of *Nicotiana benthamiana* (**a**) and *Solanum lycopersicum* (**b**) at 35 days post inoculation detected using RT-PCR. TYLCV was inoculated 7 days after foliar application with either H2O, mixed pDNA (mixed with pDNA1, pDNA2, and pDNA3), layered double hydroxide (LDH) nanosheets, or mixed pDNA–LDH (mixed with pDNA1–LDH, pDNA2–LDH, and pDNA3–LDH). Each treatment had 60 replicates. Leaf DNA was extracted from mixed leaves collected from the top, middle, and bottom of each plant. The bars on each column represent standard deviations. Values (means ± SEs) are the averages of three independent experiments, and significant differences (*P* ≤ 0.05) among treatments in each group are indicated by different letters

To determine the effect of mixed pDNA–LDH application on TYLCV prevention, the disease severity, accumulation of H_2_O_2_, and viral concentrations in TYLCV-infected *N. benthamiana* and *S. lycopersicum* were measured. H_2_O_2_ significantly increased in the leaves of the plants treated with pDNA–LDH compared to other treated plants (Fig. 5b). These results suggest that mixed pDNA–LDH application effectively protected *S. lycopersicum* against TYLCV for at least the 35-day period. Mixed pDNA–LDH treatment significantly reduced the concentration of TYLCV in the leaves of *N. benthamiana* and *S. lycopersicum* (Fig. 6a, b).

## Discussion

We have demonstrated that plasmids carrying amiRNAs were successfully delivered into plant cells with the help of LDH nanosheets and that the amiRNAs formed in the transformed plant cells were effective in suppressing the replication of TYLCV and symptom expression. The expression of amiRNA in *S. lycopersicum* leaves treated with multiple LDH-carrying pDNAs was significantly upregulated at 35 days post-TYLCV inoculation. In addition, by using multiple amiRNAs targeting different regions of the CP gene of TYLCV, the strategy reduced the risk of pathogens overcoming RNAi. LDH nanosheets were proven to be an effective carrier and delivery system for pDNA. More importantly, their slow release extended the period of effectiveness and assured long-lasting protection of plants against viral infection.

The stability of pDNA on LDH nanosheets was the key to effectively protecting and releasing pDNAs into different locations of plant cells. LDH nanosheets have been studied for carrying small RNAs and efficiently delivering them to mammalian cells in vitro^25^ and for protecting and releasing large double-stranded RNAs into plant cells^20,26^. Genomic DNA fragments of ~500–1000 bp^27^, PCR products of 800 bp^28^ and DNA molecules of 8 kb^29^ can be intercalated in the interlayer galleries of LDHs by anion exchange or a direct self-assembly route and can then be delivered into animal cells. It seems that large and sterically hindered biomolecules such as pDNAs usually adopt a supercoiled structure in solution and do not fully intercalate in the interlayer galleries by means of anion exchange^30^. In this study, we also observed a small amount of incomplete intercalation of pDNA into LDH sheets, which should not impede cellular uptake and/or protection from degradation, which is in agreement with results from Ladewig^30^.

In delivering transgenic materials into plants, pDNA has some advantages over RNA^20^. High-quality pDNA is the key to successful genetic transformation in plants. pDNA can not only be propagated in *Escherichia coli* (*E. coli*) but also be produced in large quantities through fermentation. Methods for the rapid production of large amounts of pDNA include bioprocessing, boiling^31^, a modified boiling method^32^ and alkaline lysis. Alkaline lysis is the most widely used method for extracting pDNAs from *E. coli*^33,34^. To achieve alkaline lysis at a large scale for the extraction of pDNAs from *E. coli*, Wright et al. used a simple stirred tank reactor system and increased the pDNA quantity very quickly by sequentially adding three precipitants, including a selective precipitation step with ammonium acetate^34^. This pDNA could be incorporated between hydroxide layers by a simple ion-exchange reaction to form bio-LDH nanohybrids with gentle orbital agitation, which is the simplest method of nanocomplex formation^8,25,27,35^. It is a simple, effective, easy-to-follow method for the large-scale application of pDNAs, and it exhibits slow release and does not raise biosafety concerns.

In generating amiRNAs, the choice of target site was crucial in amiRNA-mediated virus resistance^36,37^. Fortunately, it is relatively easy to pick the ideal amiRNA sequences targeting mRNAs without disturbing the expression of other genes. This feature enhances the robustness of this technology^6^. The application of multiple amiRNAs targeting the AV1/AV2 gene of TYLCV resulted in a high level of viral suppression over a certain period of time in this study (Fig. 4). This is thematically sound and promising in practice. This result is supported by results from many other studies. These studies documented effects of viral diseases such as *tomato leaf curl New Delhi virus*^37,38^, *TYLCV-Israel*^39^, *tomato leaf curl Java virus*^40^, *cotton leaf curl Burewala virus*^41^, and *bean golden mosaic virus*^42^. Multiple resistance mediated by a single pre-amiRNA was often designed using the conserved sequence of virus genes to inhibit multiple virus strains simultaneously^43–45^. In addition, multiple pre-amiRNAs concatenated under the same promoter can express multiple amiRNAs and mediate multivirus resistance in plants^46,47^. These antiviral strategies mediated by amiRNA were simple and feasible in specific experimental operations. Aragao demonstrated the feasibility of genetically engineered *Geminivirus*-resistant plants^42^. They used RNAi constructs to silence viral genes. This resulted in high resistance to viral infection, with approximately 93% of the plants being symptom free. It is important that LDH-mediated amiRNA is delivered into plants before viral infection for successful resistance. LDH nanomaterials can protect and slow the release of pDNA on the leaves for a period of time. Most likely, nanoparticle delivery in the plant cells was accomplished via natural openings—e.g., stomata (microscopic pores in leaf surfaces), hydathodes (stomata-like openings that secrete water), or plasmodesmata, which was basically consistent with the results reported by many other researchers^8,20^.

In conclusion, LDH-based pDNA is a promising tool for enhancing plant resistance against TYLCV. This method is worthy of further investigation for thorough exploitation of host-induced gene silencing against geminiviruses in future work.

## Materials and methods

### Designing and cloning of amiRNA

A schematic of the procedure for using pDNA–LDH to protect plants against *Begomovirus* is shown in Fig. S7. Tomato samples were collected in Henan Province of China. Polymerase chain reaction (PCR) was conducted using PA/PB primers and TY-FAN (+)/TY-FAN (−) reverse primers (Table S4). Three isolates of TYLCV were detected by comparing reference strains on the National Center for Biotechnology Information (NCBI, https://www.ncbi.nlm.nih.gov/) (accession numbers MF668139, MF668140, and MF668141, respectively). Sequences of the CP gene (AV1/AV2) of the three TYLCV isolates were used as query objects for the BLAST algorithm, and conserved regions containing more than 21 nucleotides were identified as target sequences. The WMD3 program (http://wmd3.weigelworld.org/cgi-bin/webapp.cgi) was used to design three corresponding amiRNAs: amiR-AV1/AV2-1 (5′-TATTATATCGCCTCGTCGCTT-3′), targeting the upstream region (7–27 bp) of the AV1 gene and the middle region (167–187 bp) of the AV2 gene; amiR-AV1-2 (5′-TTAACACAGAACCACTTACCC-3′), targeting the middle region of the AV1 gene (318–338 bp); and amiR-AV1-3 (5′-TCATATACAATAACGAGGCGT-3′), targeting the downstream region of the AV1 gene (680–700 bp).

Natural pre-miRNA (ath-miR-159a) was used as a backbone to generate three artificial pre-miRNAs (pre-amiRNAs). The sequences of pre-miR159a were replaced by those of the amiRNAs, and the whole precursors with the amiRNA sequence were synthesized and cloned into pMD19-T (simple) via *Xba* I and *Sac* I enzyme cutting sites by Sangon Biotech (Shanghai, China). Subsequently, these sequences were cut from pMD19-T (simple) with *Xba* I and *Sac* I digestion enzymes and finally transferred into the binary vector pBI121 (also cut by *Xba* I and *Sac* I) via ligation by T4 DNA ligase. Expression of all the pre-amiRNAs was driven by the CaMV 35S promoter and terminated with the NOS terminator. The clones were confirmed by DNA sequencing. Thus, three pre-amiRNAs, each containing amiR-AV1/AV2-1 (amir159-AV1/AV2-1), amiR-AV1-2 (amir159-AV1-2), or amiR-AV1-3 (amir159-AV1-3), were cloned into the pBI121 vector and used in this study. Possible folding structures of the predicted transcripts from the pre-amiRNAs were determined using the mfold program (http://mfold.rna.albany.edu/?q=mfold/RNA-Folding-Form).

### Preparation of pDNA–LDH nanosheets

LDH nanoparticles were made up of positively charged brucite-like layers, which were synthesized by a microwave-assisted aqueous coprecipitation method^24,48^. The approximate chemical formula of LDH nanomaterials was determined by energy-dispersive X-ray spectrometry (EDX-720, Shimadzu, Japan). LDH nanoparticles in suspension were characterized for size distribution and zeta potential using a Nanosizer Nano ZS (Malvern Panalytical Ltd, Malvern, UK). Morphological images of the particles were captured by TEM using a JEM-1400Plus instrument (acceleration voltage of 120 kV, JEOL, Tokyo, Japan) and a ZEISS Merlin scanning electron microscope (SEM, Carl Zeiss SMT AG, Oberkochen, Germany). White pellet-like powder was yielded by freeze-drying a concentrated suspension. The powdery product was analyzed with FTIR spectroscopy using a Brucker TENSOR 27 instrument scanning from 4000 to 400 cm^−1^ and powder X-ray diffraction using a PANalytical X’pert3 Powder X-ray diffractometer (slit width DS = 1/4°, scanning rate: 5° min^−1^, 2θ = 5°–80°, Co Kα radiation wavelength of 0.15418 nm) to verify the chemical composition and crystal structure of the nanoparticles.

pDNAs of the recombinant pBI121 vector were isolated and purified with a Qiagen Plasmid Purification Kit (Qiagen, Beijing, China). With gentle orbital agitation, three pDNAs were loaded on LDH nanosheets by electrostatic interaction. To define optimal and complete loading of pDNA into LDH nanosheets, samples were prepared whose ratios of in vitro transcribed control-pDNA (500 ng) and pDNA (1 µg) to LDH (pDNA–LDH (w/w)) were 1:1, 1:2, 1:4, 1:8, 1:10, 1:12, and 1:16. A 10 µl aliquot of pDNA and different proportions of LDH nanosheets were incubated at room temperature (25 °C) for 30 min with gentle orbital agitation. Complete pDNA loading was assessed by retention of pDNA–LDH complexes in wells of a 1% agarose gel. Three pDNA–LDH nanosheets were separately prepared, and the morphologies were imaged with TEM.

### Stability of pDNA on LDH nanosheets

To examine the stability of pDNA loaded on LDH nanosheets, acidic and enzymatic treatments were applied. pH changes were monitored by dissolving LDH nanosheets under strongly acidic conditions by adding 5 ml of 2 mM HNO_3_ (pH 3.0) to 5 ml of 1 µg/µl LDH nanosheets. pH values were measured at 1, 3, 10, 20, 30, and 60 min after treatment. The release of pDNA from the pDNA–LDH complexes was determined at different pH values. Control-pDNA–LDH (1:10, 10 µl) was first precipitated by adding 30 µl of 1 M NaCl for 20 min. The precipitate was pelleted by centrifugation at 9000 × *g* for 30 min. Pellets were resuspended in 10 µl solutions (pH 2.0–12.0, adjusted with either HNO_3_ or NaOH) and subjected to 1% agarose gel electrophoresis within 5 min of resuspension. Since LDH nanosheet-bound pDNA did not migrate, fluorescence could be observed in the well.

The ability of LDH nanosheets to protect loaded pDNA from potential environmental breakdown was investigated by the exposure of pDNA (500 ng) and pDNA–LDH (500 ng/2.5 µg) to DNase I (TaKaRa, Dalian, China) treatments. Samples were treated with 70 U of DNase I for 4 min at 25 °C. pDNA was released by acidic dissolution of LDH nanosheets using release buffer (4.11 ml of 0.2 M Na_2_HPO_4_ + 15.89 ml of 0.1 M citric acid; pH 3) at a pDNA–LDH buffer volume ratio of 5:1. Nuclease-treated and untreated samples were examined on a 1% agarose gel.

### Uptake and transfection of pDNA in plant cells

To detect pDNA uptake by plants and consequent movement in plant cells, three pDNAs were labeled with 1,1P-(4,4,8,8, tetramethyl-4,8-diazaundecamethylene) bis[4- [3-methyl-benzo-1,3-oxazol-2-yl] methylidene]-1,4-dihydroquinolinium] tetraiodide (YOYO-1) (Invitrogen, Shanghai, China), a green fluorescent dye for visualizing DNA molecules, per the manufacturer’s recommendations and then loaded on LDH nanosheets. The labeled pDNA–LDH nanosheets were sprayed on the inner surface of onion and the leaf surface of *N. benthamiana*. To visualize nuclei, epidermis of onion and leaves of *N. benthamiana* were stained with 4′,6-diamidino-2-phenylindole (DAPI, 5 mg/mL, Sigma-Aldrich, St. Louis, USA). The plant tissues were soaked in phosphate buffer solution (PBS) containing DAPI (pH 7.0; DAPI:PBS (v/v) = 1:1000) and kept in darkness for 20 min. The soaked tissues were washed with PBS three times before observation. The samples were examined under a Zeiss LSM780 META confocal laser scanning microscope (Carl Zeiss, Jena, Germany) after 72 h.

### amiRNA expression analysis

*N. benthamiana* and *S. lycopersicum* “Moneymaker” were used as hosts for amiRNA expression. All plants were grown in potting soil in 10 cm-wide pots under greenhouse conditions (25 °C, 70% relative humidity) unless otherwise specified. At the three-leaf stage for *N. benthamiana* and two-leaf stage for *S. lycopersicum*, plants were sprayed with pBI121-LDH, pDNA1-LDH (amiRNA-AV1/AV2-1), pDNA2-LDH (amiRNA-AV1-2), pDNA3-LDH (amiRNA-AV1-3), or pDNA(1 + 2 + 3)-LDH (mixed with three pDNA-LDHs). The loading ratio of the five pDNA–LDH nanosheets was 1:10. Plants were sprayed with an atomizer at ~125 µl/cm^2^ leaf surface.

qRT-PCR assays were performed to evaluate the expression levels of mixed mature miRNAs as previously described after 7 days. Total RNA and small RNA fractions of leaves of *N. benthamiana* and *S. lycopersicum* were extracted using a TRIzol (Life Technologies, USA)-based method following the manufacturer’s protocol. Expression of mixed miRNAs was quantified by qPCR using a Mir-X™ miRNA First-Strand Synthesis kit (TaKaRa Bio, USA), which is a complete, dual function system for performing first-strand cDNA synthesis and qPCR to precisely measure the level of miRNAs. The three upstream primers were miRNA-specific primers, which are described in Table S4. The downstream primers were the mRQ 3′ primer provided in the kit. The results were normalized to the U6 gene of *N. benthamiana* and *S. lycopersicum* (the primer sequences amplifying the U6 gene are shown in Table S4). Mixed samples were used in qRT-PCR analysis, and three biologically independent replicates were analyzed for each sample. Plants sprayed with pBI121-LDH were used as controls. Data were analyzed using the 2^−ΔΔCt^ method.

### Foliar spray with pDNA–LDH for TYLCV prevention

#### Plant inoculation with TYLCV

*N. benthamiana* and *S. lycopersicum* “Moneymaker” were used as hosts for symptom expression. At the three-leaf stage for *N. benthamiana* and two-leaf stage for *S. lycopersicum*, plants were sprayed with either H_2_O (distillation–distillation H_2_O), LDH, pDNA(1 + 2 + 3) (mixture of three pDNAs), or pDNA(1 + 2 + 3)-LDH. The loading ratio of pDNA1, pDNA2, and pDNA3 to nanosheets in the pDNA–LDH complex was 1:10. H_2_O, pDNA (1:0), and LDH (0:3) were used as controls. The leaf surfaces of plants were sprayed with 1.25 µg of pDNA, 3.75 µg of LDH, or H_2_O using an atomizer at ~125 µl/cm^2^. Leaf cells and stomates of *N. benthamiana* and *S. lycopersicum* plants were observed 30 min after foliar spraying to evaluate the effects of treatment. Seven days later, viral challenging assays were carried out in the sprayed plants using an agro-infectious clone of TYLCV-[CN:SH2] (GenBank accession number: AM282874). Plants were kept at 25 °C with 70% relative humidity and a 16 h/8 h photoperiod in an insect-free greenhouse, and symptom development was examined daily.

#### Detection of H2O2 in plants

TYLCV-inoculated leaves were subjected to 3,3-diaminobenzidine (DAB) staining^49^ to investigate the accumulation of H_2_O_2_ and its production location. Leaves of *N. benthamiana* and *S. lycopersicum* were excised from the plant and placed in 20 mL tubes, covered with DAB-HCl solution (1 mg/ml, pH 3.8) and incubated in a growth chamber for 8 h at 25 °C. Once red-brown DAB solution moved up to the top of leaves along the veins, chlorophyll from whole leaves was removed by directly immersion into a fixing solution (anhydrous ethanol:acetic acid = 3:1) for 24 h. The stained leaves were processed with hydrated trichloroacetaldehyde three times and observed using an optical microscope.

#### Delivery of pDNA

To determine whether pDNAs were delivered into plant leaves, PCR was conducted on unsprayed new leaf samples of both *N. benthamiana* and *S. lycopersicum*. Total DNA of leaves was extracted using a plant genomic DNA extraction kit (TaKaRa, Dalian, China) following the manufacturer’s protocol. The primers pBI121-F/pBI121-R (Table S4) were used to amplify the pBI121 vector.

#### Detection of TYLCV using qPCR

Expression of TYLCV was quantified by targeting leaves from mixed leaves collected from the top, middle, and bottom of plants by qPCR using SYBR Premix Ex Taq (TaKaRa) on an ABI Prism 7500 thermal cycler (Applied Biosystems, Waltham, USA) with the primer pairs Nb-GAPDH-F/Nb-GAPDH-R, TYLCV-YG-3/TYLCV-YG-4, and Tomato25s-Rrna-UNIV (+)/Tomato25s-Rrna-UNIV (−) (Table S4). Triplicate PCRs were conducted for each DNA sample, and the assay consisted of three technical replicates. Data were analyzed using the 2^−ΔΔCt^ method.

#### Detection of TYLCV using ELISA analysis

TYLCV was detected from mixed leaves collected from the top, middle and bottom of plants using antigen-coated plate (ACP)-ELISA according to standard methods. The antibody used in ACP-ELISA was reacted against the CP of TYLCV. The reactions were followed by measuring the optical density at 405 nm on a spectrum plate reader (Thermo Fisher Scientific, Waltham, USA). A sample was considered TYLCV positive when the absorbance values were two times higher than those of noninoculated plant samples.

## Supplementary information

Supplemental figures and tables-R2
