# Supplementary material for: Clay nanosheet-mediated delivery of recombinant plasmids expressing artificial miRNAs via leaf spray to prevent infection by plant DNA viruses
Source: Hortic Res. 2020 Nov 1;7:179. doi: 10.1038/s41438-020-00400-2 (PMC7603507; doi:10.1038/s41438-020-00400-2)
Supplement: Supplementary file 1 — Supplemental figures and tables-R2 [file 41438_2020_400_MOESM1_ESM.docx]

**Supplemental figures and tables**

**Fig. S1**

**B**

**A**


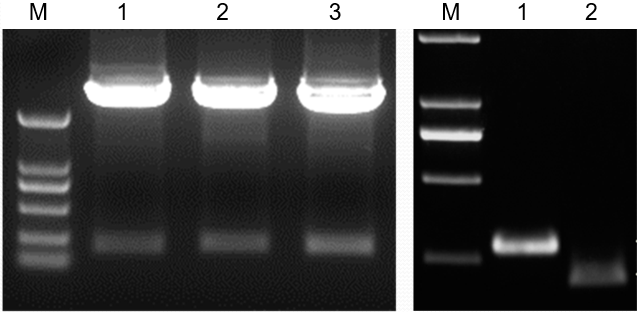


2000bp

250bp

100bp

293bp

196bp

**Fig. S2**


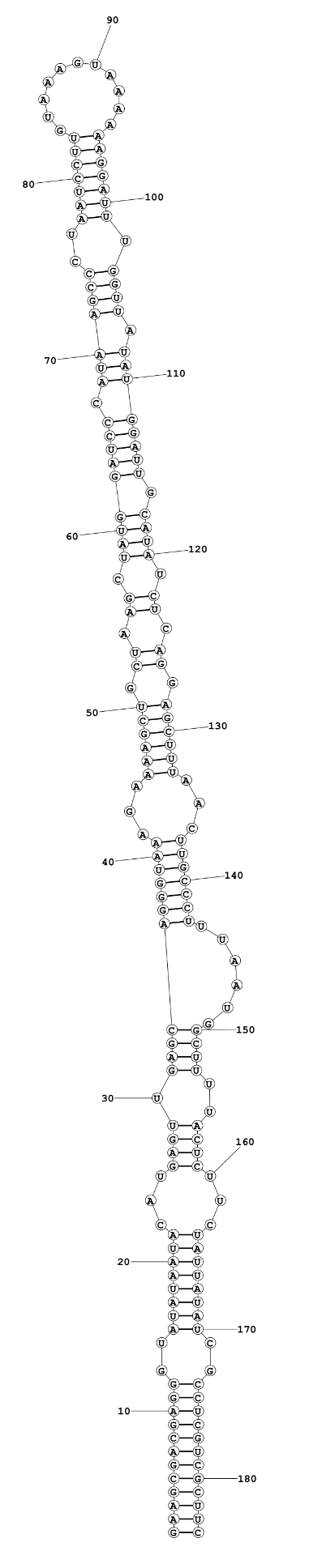

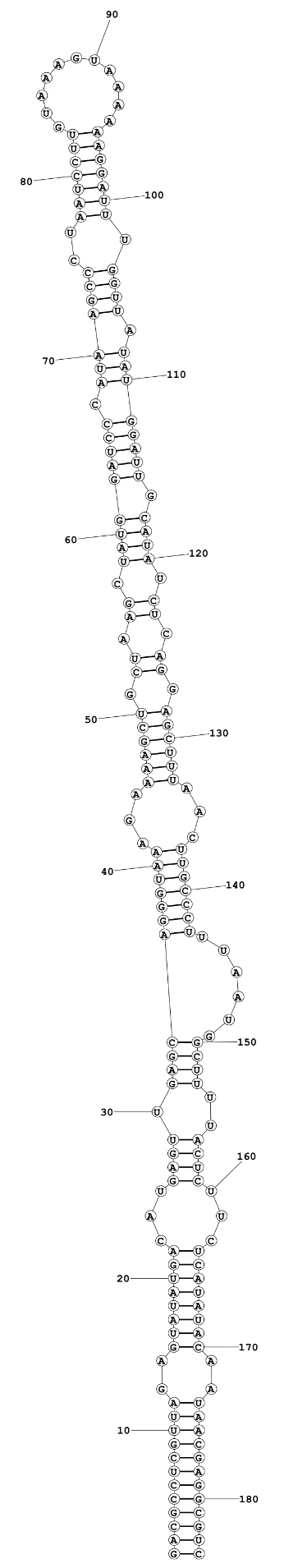

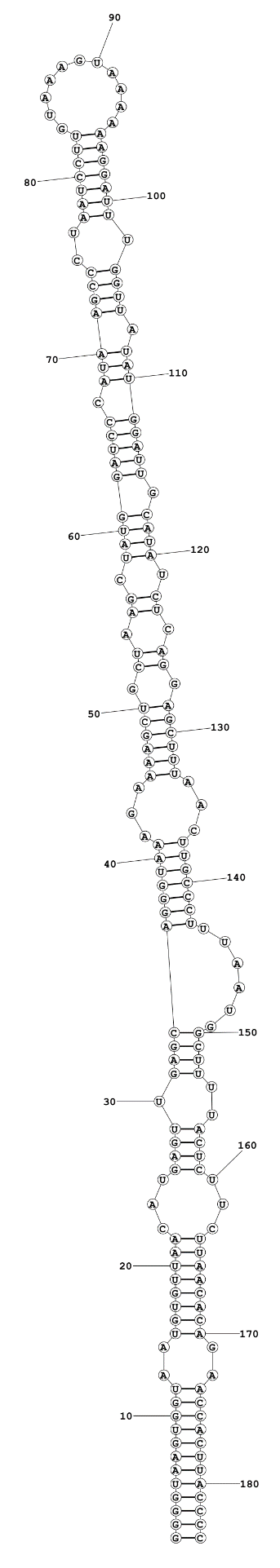

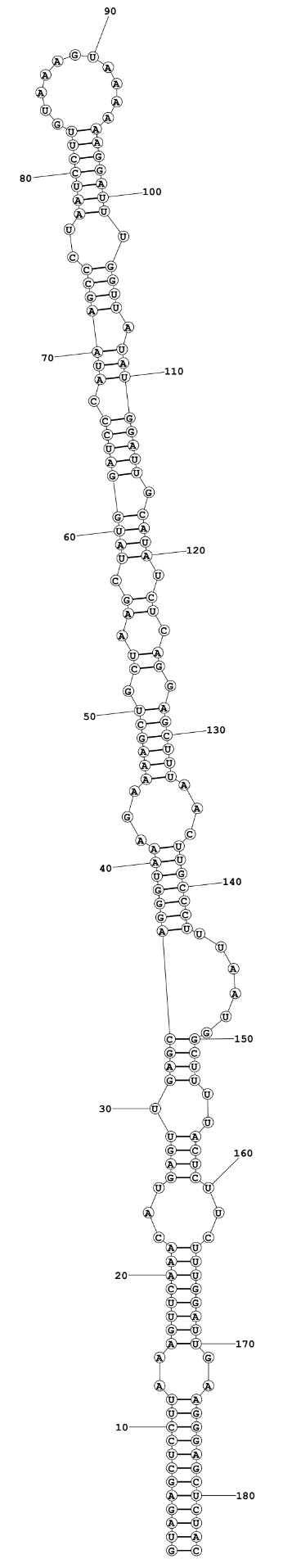


amiRNA

A B C D

**Fig. S3**

| element | weight % | content |
| --- | --- | --- |
| C | 8.48 | 12.34 |
| N | 1.69 | 2.10 |
| O | 58.10 | 63.47 |
| Mg | 21.88 | 15.73 |
| Al | 9.71 | 6.29 |
| Cl | 0.14 | 0.07 |
| In total | 100.00 | 100.00 |


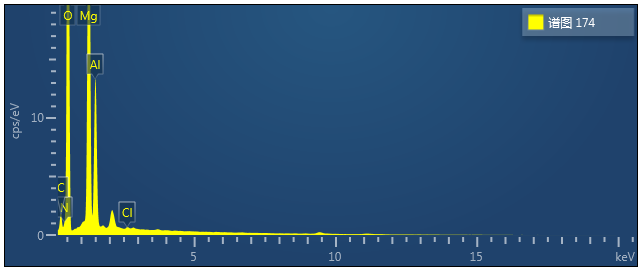

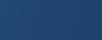


**Fig. S4**







**A**


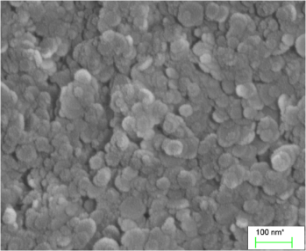

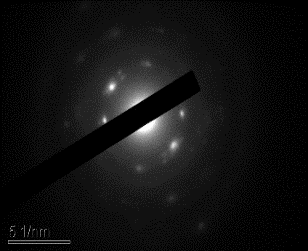

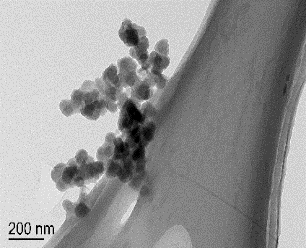


**D**

**C**

**E**

**B**

**Fig. S5**


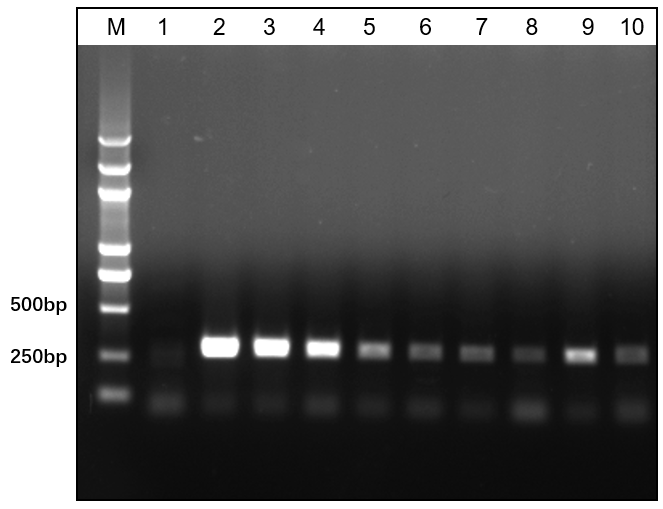


**Fig. S6**

**A**


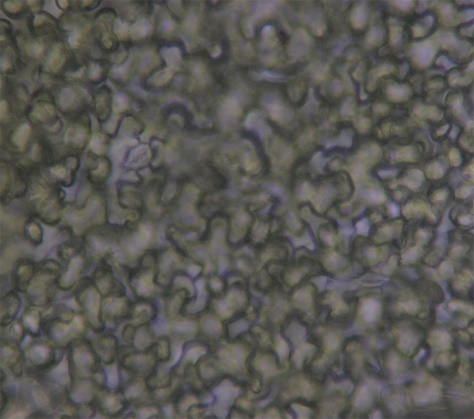

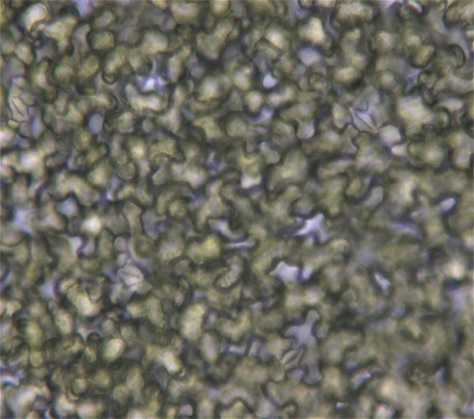

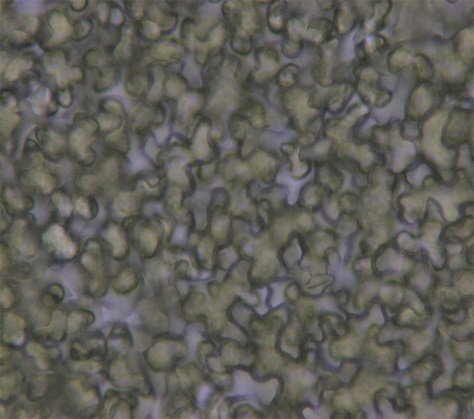


**Left:**

**pDNA**

**Right:**

**pDNA-LDH**

**A-1**

**A-2-pDNA**

**A-2**

**A-2-pDNA-LDH**


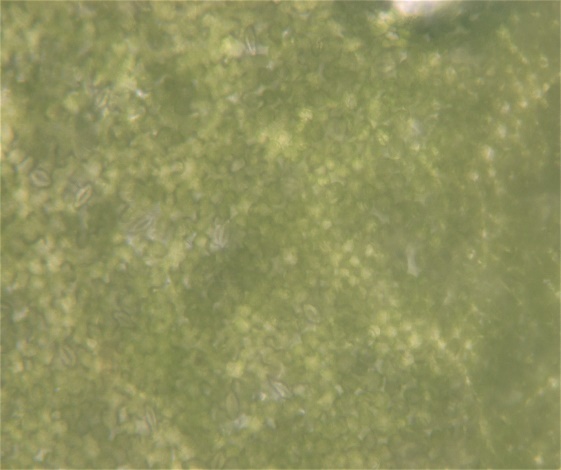


**B-1**

**B-2**

**B**


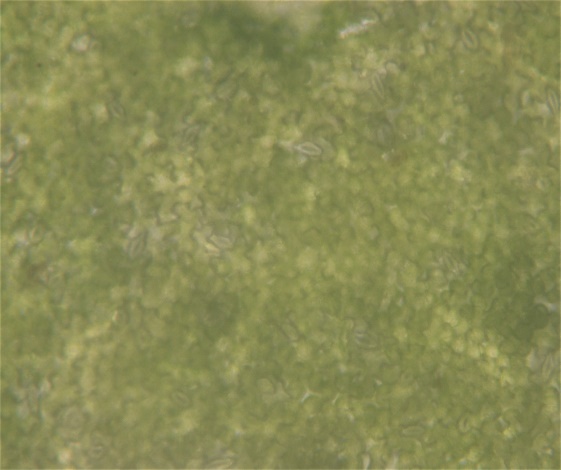


**B-2-pDNA**


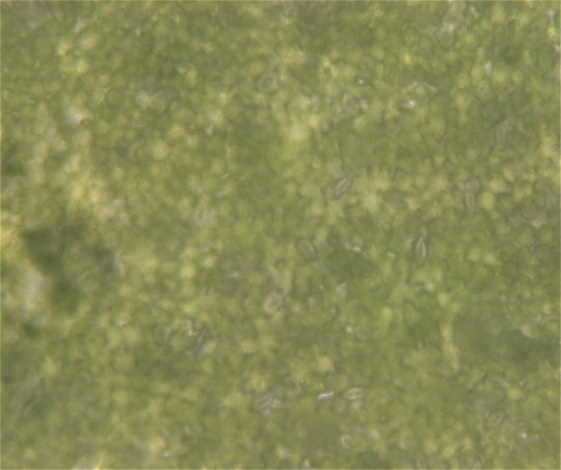


**B-2-pDNA-LDH**

**Right:**

**pDNA-LDH**

**Left:**

**pDNA**

**Fig. S7**

**
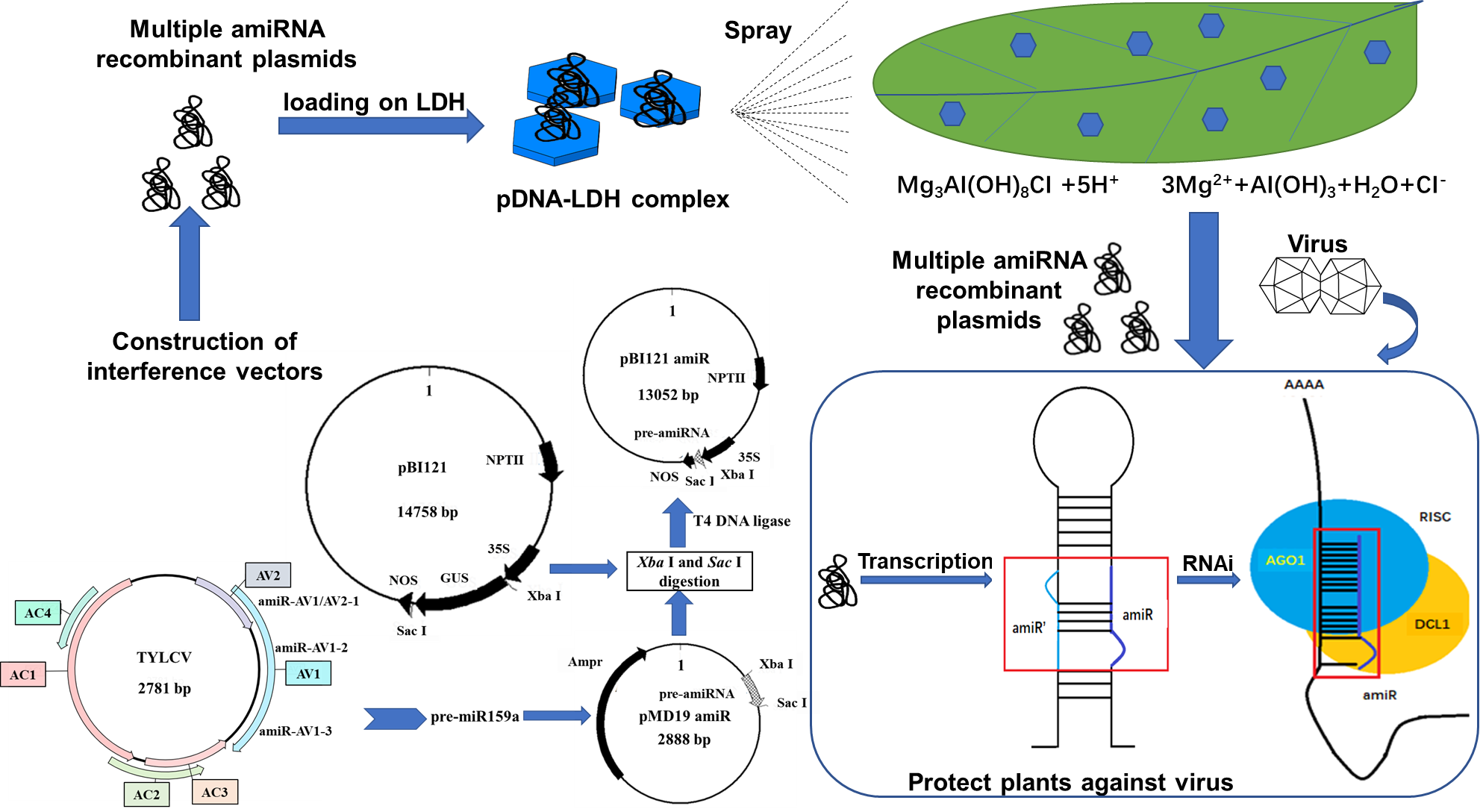
**

**Table S1**. Sequences and positions in the target transcripts of artificial microRNA (amiRNA)

| Name of amiRNA | Sequence (5’–3’) | Target position (nucleotide # from 5’ end) |
| --- | --- | --- |
| amiR-AV1/AV2-1 | UAUUAUAUCGCCUCGUCGCUU | Upstream region (7 to 27 bp) of AV1 transcript and middle region (166 to 186 bp) of AV2 transcript |
| amiR-AV1-2 | UUAACACAGAACCACUUACCC | Middle transcript (318 to 338 bp) of AV1 |
| amiR-AV1-3 | UCAUAUACAAUAACGAGGCGU | Downstream transcript (680 to 700 bp) of AV1 |

**Table S2** Infectivity of *Tomato yellow leaf curl virus* (TYLCV) in *Nicotiana benthamiana* (N. b.) and *Solanum lycopersicum* (S. l.) plants spraying multiple amiRNA recombinant plasmids loaded on LDH at 35 days post TYLCV inoculation

| Plants | Symptomatic plants /total plants | | RT-PCR^a^ | | ELISA^b^ | |
| --- | --- | --- | --- | --- | --- | --- |
|  | N. b. | S. l. | N. b. | S. l. | N. b. | S. l. |
| H_2_O + TYLCV | 48/55 | 49/55 | 48/55 | 49/55 | ++++ | ++++ |
| LDH + TYLCV | 40/50 | 48/50 | 40/50 | 49/50 | ++++ | ++++ |
| pDNA + TYLCV | 39/54 | 38/54 | 40/54 | 39/54 | +++ | +++ |
| pDNA-LDH + TYLCV | 28/60 | 25/60 | 31/60 | 27/60 | ++ | + |

^a^TYLCV was detected DNAs extracted from plants by RT-PCR.

^b^ ELISA assay results ([average](javascript:;) [value](javascript:;)) were given as strong (++++), through weak (+) to no detected (-).

**Table S3.** Pre-amiRNA used in this study

| Name | Sequence（5’ to 3’） |
| --- | --- |
| Pre-amiRNA-1 | TCTAGAGAAGCGACGAGGGTATATAATACATGAGTTGAGCAGGGTAAAGAAAAGCTGCTAAGCTATGGATCCCATAAGCCCTAATCCTTGTAAAGTAAAAAAGGATTTGGTTATATGGATTGCATATCTCAGGAGCTTTAACTTGCCCTTTAATGGCTTTTACTCTTCTATTATATCGCCTCGTCGCTTCGAGCTC |
| Pre-amiRNA-2 | TCTAGAGGGGTAAGTGGTAATGTGTTAACATGAGTTGAGCAGGGTAAAGAAAAGCTGCTAAGCTATGGATCCCATAAGCCCTAATCCTTGTAAAGTAAAAAAGGATTTGGTTATATGGATTGCATATCTCAGGAGCTTTAACTTGCCCTTTAATGGCTTTTACTCTTCTTAACACAGAACCACTTACCCC GAGCTC |
| Pre-amiRNA-3 | TCTAGAGACGCCTCGTTAGAGTATATGACATGAGTTGAGCAGGGTAAAGAAAAGCTGCTAAGCTATGGATCCCATAAGCCCTAATCCTTGTAAAGTAAAAAAGGATTTGGTTATATGGATTGCATATCTCAGGAGCTTTAACTTGCCCTTTAATGGCTTTTACTCTTCTCATATACAATAACGAGGCGTC GAGCTC |

**Table S4.** Primers used for virus, vector and amiRNA detection in conventional PCR and quantitative real-time fluorescence PCR (qRT-PCR)

| Primer name | Primer sequence (5’-3’) | Size of PCR products (bp) | Application |
| --- | --- | --- | --- |
| **PA** | **TAATATTACCKGWKGVCCSC** | **550** | **Detect Geminivirus fragment by conventional PCR** |
| **PB** | **TGGACYTTRGAWGGBCCTTCACA** |  |  |
| TY-FAN (+) | TACAGAATGTATCGAAGC | 2300 | Reverse primers for detect the other fragment of Geminivirus by conventional PCR |
| TY-FAN(-) | TCTAACGTTTGAG GATGC |  |  |
| **pBI 121-F** | **AAGGAAGTTCATTTCATTTG** | **300** | **Detect pBI121 by conventional PCR** |
| **pBI 121-R** | **GCAACAGGATTCAATCTTAA** |  |  |
| TYLCV-YG-3 | GAGTTCCCCTGTGCGTGA | 139 | Detect TYLCV by qRT-PCR |
| TYLCV-YG-4 | CTGTTCGCAAGTATCAATCAAGGT |  |  |
| **Nb-GAPDH-F** | **AGCTCAAGGGAATTCTCGATG** | **125** | **Detect GAPDH gene of *Nicotiana benthamiana* by qRT- PCR** |
| **Nb-GAPDH-R** | **AACCTTAACCATGTCATCTCCC** |  |  |
| Tomato25s-Rrna-UNIV (+) | ATAACCGCATCAGGTCTCCA | 113 | Detect tomato 25s rRNA by qRT-PCR |
| Tomato25s-Rrna-UNIV (-) | CCGAAGTTACGGATCCATTT |  |  |
| **Q-amiR-amiR-AV1/AV2-1** | **TATTATATCGCCTCGTCGCTT** |  | **miRNA specific 5’ primer used in qPCR (The 3’ primer is supplied with Mir-X™ miRNA first-strand synthesis kit (Takara Bio, USA).** |
| **Q-amiR-AV1-2** | **TTAACACAGAACCACTTACCC** |  |  |
| **Q-amiR-AV1-3** | **CTCATATACAATAACGAGGCGT** |  |  |
| U6-F | GGAACGATACAGAGAAGATTAGC | 65 | Detect U6 gene of ***Solanum lycopersicum*** and ***Nicotiana benthamiana*** by qRT- PCR |
| U6-R | CCATTTCTCGATTTGTGCG |  |  |
